# Influence of Polypropylene Fiber on Concrete Permeability under Freeze-Thaw Conditions and Mechanical Loading

**DOI:** 10.3390/ma17122945

**Published:** 2024-06-15

**Authors:** Wei Zeng, Weiqi Wang, Qiannan Wang, Mengya Li, Lining Zhang, Yunyun Tong

**Affiliations:** 1 School of Civil Engineering and Architecture, Zhejiang University of Science and Technology, Hangzhou 310023, China; wei-zeng@zust.edu.cn (W.Z.); 121032@zust.edu.cn (W.W.); wangqiannan@zust.edu.cn (Q.W.); 2 Zhejiang International Science and Technology Cooperation Base for Waste Resource Recycling and Low-Carbon Building Materials Technology, Zhejiang University of Science and Technology, Hangzhou 310023, China; 3 L2MGC, EA 4114, CY Cergy Paris Université, 95000 Cergy, France; mengya.li1@cyu.fr; 4 Department of Mechanical and Mechatronics Engineering, The University of Auckland, Auckland 1010, New Zealand; lzha977@aucklanduni.ac.nz

**Keywords:** polypropylene fiber, permeability, freeze-thaw damage, tensile load, compressive load, concrete structure

## Abstract

Polypropylene fiber reinforcement is an effective method to enhance the durability of concrete structures. With the increasing public interest in the widespread use of polypropylene fiber reinforced concrete (PFRC), the necessity of evaluating the mechanism of polypropylene fiber (PF) on the permeability of concrete has become prominent. This paper describes the influence of PF on the concrete permeability exposed to freeze-thaw cycles under compressive and tensile stress. The permeability of PFRC under compressive and tensile loads is accurately measured by a specialized permeability setup. The permeability of PFRC under compressive and tensile loads, the volume change of PFRC under compressive load, and the relationship between compressive stress levels at minimum permeability and minimum volume points of PFRC are discussed. The results indicate that the addition of PF adversely affects the permeability of concrete without freeze-thaw damage and cracks. However, it decreases the permeability of concrete specimens exposed to freeze-thaw cycles and cracking. Under compressive load, the permeability of PFRC initially decreases slowly and follows by a significant increase as the compressive stress level increases. This phenomenon correlates with the volume change of the specimen. The compressive stress level of the minimum permeability point and compressive stress level of the minimum volume point of PFRC exhibit a linear correlation, with a fitted proportional function parameter *γ* ≈ 0.98872. Under tensile load, the permeability of PFRC increases gradually with radial deformation and follows by a significant increase. The strain-permeability curves of PFRC under loading are studied and consist of two stages. In stage I, the permeability of PFRC gradually decreases with the increase of strain under compressive load, while the permeability increases with the increase of strain under tensile load. In stage II, under compressive load, the permeability of PFRC increases with the increase of freeze-thaw cycles, whereas under tensile load, the permeability gradually decreases with the increase of freeze-thaw cycles. The reduction of PF on the permeability of PFRC under tensile load is greater than that under compressive load. In future research, the relationship between strain and permeability of PFRC can be integrated with its constitutive relationship between stress and strain to provide a reference for the application of PF in the waterproofing of concrete structures.

## 1. Introduction

In cold regions, freeze-thaw damage is the primary cause of concrete deterioration. Concrete exposed to freeze-thaw cycles often exhibits increased internal micro-cracks, thus resulting in reduced load-bearing capacity. The appearance of micro-cracks in concrete matrix increases its permeability. Elevated permeability can amplify corrosion by external agents, thus reducing the service life of concrete structures [1,2]. Current research suggests that the addition of polypropylene fiber (PF) as reinforcement can improve the permeability resistance of concrete [1,2,3,4]. 

The PF significantly restricts the width of macro-cracks induced by loading and freeze-thaw cycles [5]. It effectively increases the tortuosity and surface roughness of the cracks. This leads to the great head loss and substantially reduced permeability of the cracks in concrete structures [6,7]. Additionally, Zhou et al. [8] have demonstrated that PF significantly decreases the release rate of elastic strain energy in concrete, thereby increasing the dissipation energy. This enhancement in energy dissipation improves the ductility and toughness of the concrete, reducing its brittleness and crack width under load, and consequently decreasing the permeability of cracks under loading [9,10]. However, Zhu et al. [11] have found that the inclusion of PF reduces the workability of fresh concrete and increases the connectivity of pores in the hardened concrete matrix, thereby enhancing the permeability of concrete matrix. Therefore, studying the effect of PF on the permeability of concrete under freeze-thaw conditions is necessary to evaluate the effects of the concrete matrix and cracks on the permeability of concrete structures. In order to study the effect of PF on the permeability of concrete under freeze-thaw conditions, it is necessary to study and evaluate the influence of concrete matrix and concrete crack on the concrete permeability, respectively.

Many countries and regions have specified codes for concrete permeability, including China (GB/T 50082-2009) [12], the US (ASTM C1202) [13], and Europe (BS EN 12390-8) [14]. Consequently, based on the above specified codes, many studies have focused solely on the permeability of concrete matrix. However, the concrete structure works with cracks [15,16], and the matrix permeability is much lower than the permeability of cracks [17,18]. Using the impermeability of sound concrete as a benchmark can result in an overestimation of its impermeability. Conversely, using only the crack permeability as a criterion might result in an underestimation of the permeability. To ensure an accurate assessment of concrete permeability during the service stage, the permeability of both the concrete matrix and the crack must be evaluated. However, there is limited research on how PF affects the permeability of concrete subjected to freeze-thaw cycles under loading.

Since most concrete structures in engineering operate under load, researchers including Akhavan et al. [19] and Padilla et al. [20] have induced cracks in concrete samples through loading and subsequently tested the permeability of these cracked specimens after unloading. Meanwhile, Rastiello et al. [21], Choinska et al. [22], and Wang et al. [23] have utilized customized permeability testing tools to perform experiments under constant load conditions. However, concrete permeability varies under different loads. In numerous engineering applications, concrete structures predominantly undergo bending, which results in both compressive and tensile loads [16,24]. Studies regarding the concrete permeability under compressive and tensile loads are scarce. Therefore, concrete permeability testing equipment must be developed to measure permeability under different loads. This allows further investigation of the influence of PF on the concrete permeability subjected to freeze-thaw cycles under various loading conditions. 

Therefore, this study was performed to investigate the effect of PF on the concrete permeability exposed to freeze-thaw cycles under compressive and tensile loads. A series of experiments was carried out: (a) a rapid freeze-thaw test was conducted to induce freeze-thaw damage on polypropylene fiber reinforced concrete (PFRC) specimens; (b) permeability tests under compressive and tensile loads were performed to assess the permeability; (c) volume changes under compressive loads were analyzed to verify the relationship between the permeability of concrete and the volume of hollow cylindrical specimens; and (d) strain-permeability curves of PFRC under compressive and tensile loads were analyzed. This study offers both empirical data and theoretical insights for promoting the use of PFRC for impermeability projects in freeze-thaw environments.

## 2. Experiments

### 2.1. Materials

**Figure 1:**
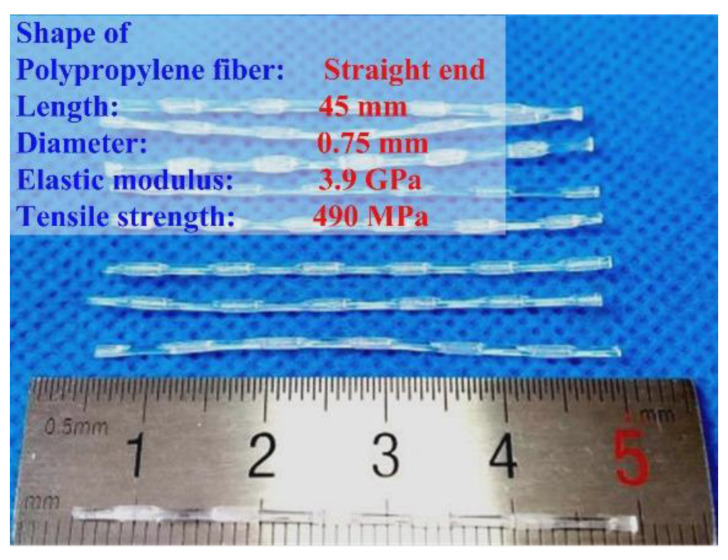
Properties and geometry of polypropylene fiber (the blue texts are the parameter names of PF and the red texts are the parameter values of PF).

In this study, P.O 42.5R Portland cement and fly ash were used. The fine aggregate was quartz sand with a fineness modulus of 2.6 and a particle size between 0 and 5 mm. The coarse aggregate was natural crushed gravel with particle size between 5 and 10 mm. Typically used polypropylene fiber was incorporated into the concrete specimens. Figure 1 shows the properties and geometry of the PF. Mix proportions of PFRC and NC samples are shown in Table 1. 

With the increase in PF content, the workability of fresh PFRC reduces. Thus, the PF content of PFRC is mainly restricted by the workability of fresh PFRC. Based on previous studies, PF content below 0.75 vol.% (equivalent to 6.9 kg/m^3^) ensures good workability of fresh concrete [25,26,27]. Therefore, the PF content in PFRC specimens does not exceed 0.75 vol.% in this study. In engineering practice, the mix proportion of concrete is commonly expressed in mass per cubic meter (kg/m^3^) [28]. Therefore, in this study, the PF mass dosages of PFRC specimens were 2.3 kg/m^3^ (0.25 vol.%), 4.6 kg/m^3^ (0.50 vol.%), and 6.9 kg/m^3^ (0.75 vol.%), respectively. The normal concrete (NC) specimen was used as the reference specimen.

### 2.2. Test Specimens

**Figure 2:**
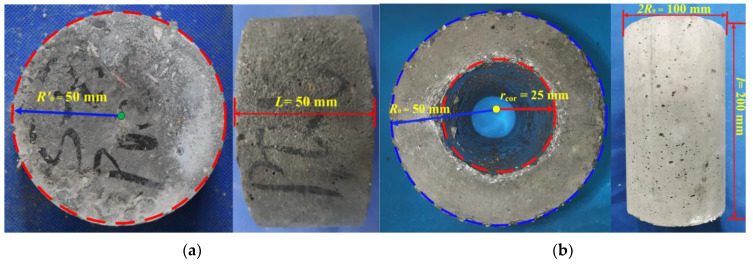
Concrete specimens: (**a**) cylindrical specimen, (**b**) hollow cylindrical specimen (The red and blue dashed lines are the outline lines of concrete specimens, the green and yellow dots are the center points of concrete specimens, and the yellow texts are the size parameters of concrete specimens).

In this study, both cylindrical and hollow cylindrical specimens of PFRC and NC were prepared. Fresh concrete was cast in a steel mold with size: Depth × Width × Length = 200 mm × 400 mm × 400 mm, which was de-molded after 1 day and cured in a standard curing room for 28 days. The cylindrical concrete specimens (size: radius = 50 mm and height = 200 mm) were obtained from the hardened concrete using a core drilling machine. Then, a cutting machine was used to cut cylindrical concrete specimens (size: radius *R*′_0_ = 50 mm and height *L* = 50 mm), which were used to estimate the concrete permeability under tensile load, as depicted in Figure 2a.

Simultaneously, a hollow-core concrete region with a diameter of 50 mm was obtained along the axis of the 200-mm-high cylindrical concrete specimens using the core drilling machine. Through this process, hollow cylindrical specimens (size: outer radius *R*_0_ = 50 mm, inner radius *r_cor_* = 25 mm, and height *l* = 200 mm) were produced and used to evaluate the concrete permeability under compressive load. For each concrete mixture, a minimum of six cylindrical specimens waere prepared for permeability tests under tensile load, as depicted in Figure 2b. Additionally, six hollow cylindrical specimens were prepared for permeability tests under compressive load and another six hollow cylindrical specimens were prepared for compressive volume deformation tests.

### 2.3. Rapid Freeze-Thaw Test

**Figure 3:**
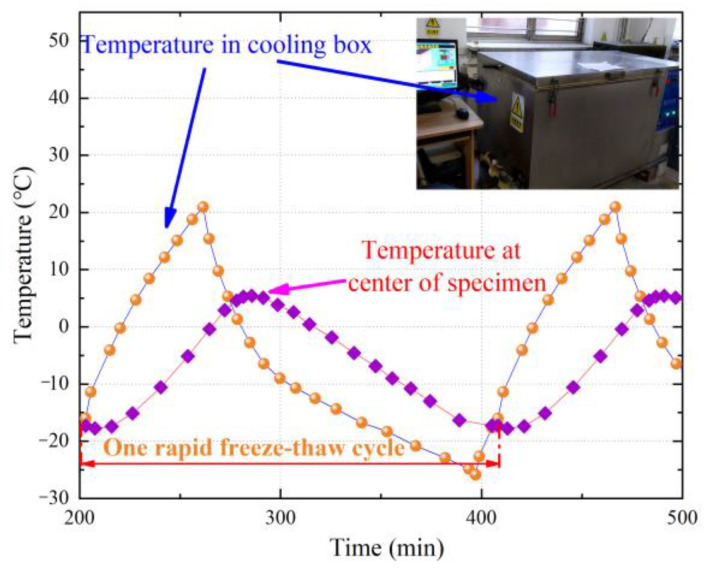
Time-temperature curves (the purple square line is the temperature-time curve at center of specimen, and the orange circle line is the temperature-time curve in cooling box).

Concrete specimens from each group were subjected to rapid freeze-thaw cycles in accordance with ASTM C666 [29]. Figure 3 shows the time-temperature curves of both the temperature control chamber and the central concrete specimen. The target freeze-thaw cycles for each type of specimen were 0, 50, 100, and 150. In this study, the specimens were named according to the PF content and the number of freeze-thaw cycles. For example, a PP2.3 specimen subjected to 50 freeze-thaw cycles is referred to as “PP2.3-50”.

### 2.4. Permeability Test

**Figure 4:**
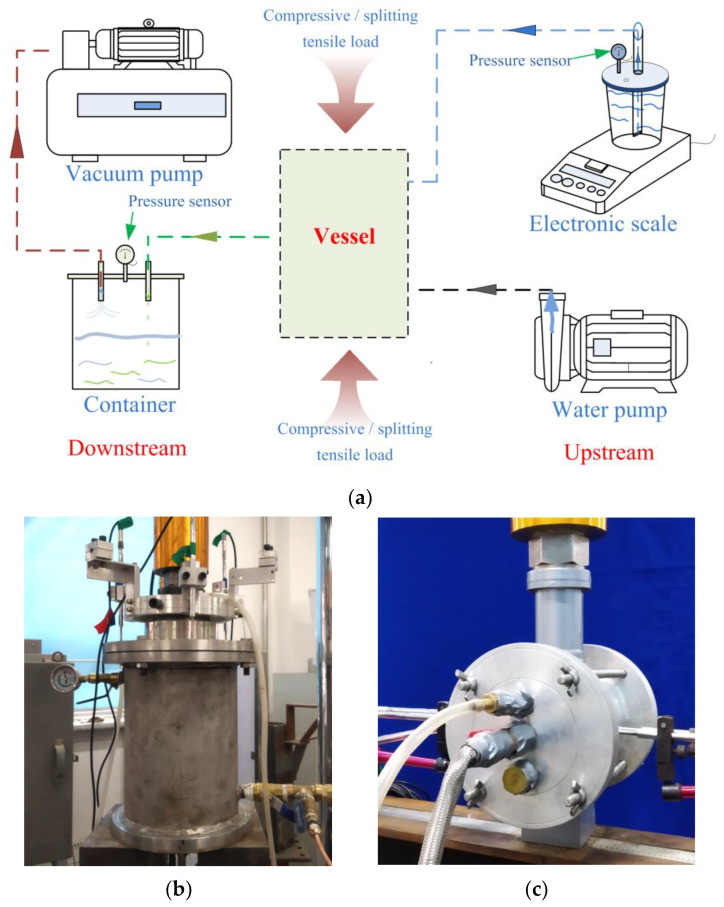
(**a**) Schematic illustration of permeability setup; Vessel under (**b**) compressive load and (**c**) tensile load.

Inspired by the experimental approach of Rastiello et al. [20], a new permeability testing apparatus was developed. Figure 4a illustrates the principle of the device. The vacuum pump reduces the air pressure on the water outflow side, thus establishing a pressure gradient between the upstream and downstream. This differential pressure prompts water transfer from the beaker to the concrete specimen. The instantaneous water flow rate and permeability of the specimen can be deduced by monitoring the real-time decrease in water mass in the beaker using a mass sensor with an accuracy of 0.001 g. This technique can resolve the delay caused by water flowing through the conduit and thus improve the accuracy of the test results.

The permeability testing apparatus generates a 10 kPa pressure difference (Δ*P*) between the upstream and downstream by the vacuum pump. Prior to loading, a 4-h permeability test was performed on the specimen to ensure stable seepage. An MTS 250 kN hydraulic servo testing machine was used, and a displacement loading rate of 0.01 mm/min was adopted to facilitate controlled crack development.

Figure 4b,c illustrate the experimental vessels for the permeability tests under both compressive and tensile loads. The tensile load on the PFRC was provided by the splitting tensile test. Flexible waterproof tape was applied to the non-permeable areas of the specimens to inhibit seepage. The water pressure gradient in the permeability test remained below 1.8 MPa/m, ensuring the laminar flow through the concrete specimen. Darcy’s law was used to evaluate the concrete permeability performance [30,31]. Permeability of concrete *κ* under compressive and tensile loads can be calculated using Equations (1) and (2), respectively.

(1) $$\kappa_{C}=\frac{Q\mu }{2\pi l_{0}\Delta P}\ln\frac{R_{0}}{r_{\mathrm{cor}}}$$

(2) $$\kappa_{T}=\frac{Q}{A_{B}}\frac{\mu }{\rho }{\left(\frac{△p}{△x}\right)}^{-1}$$

where *κ*_C_ is the permeability under compressive load (m^2^), *κ*_T_ is the permeability under tensile load (m^2^), *µ* is the dynamic viscosity of water, *Q* is the water flow rate (m^3^/s), and *l*_0_ is the height of the permeable area of hollow cylindrical specimen (0.179 m).

### 2.5. Measurement of Volume Change of PFRC under Compressive Load

**Figure 5:**
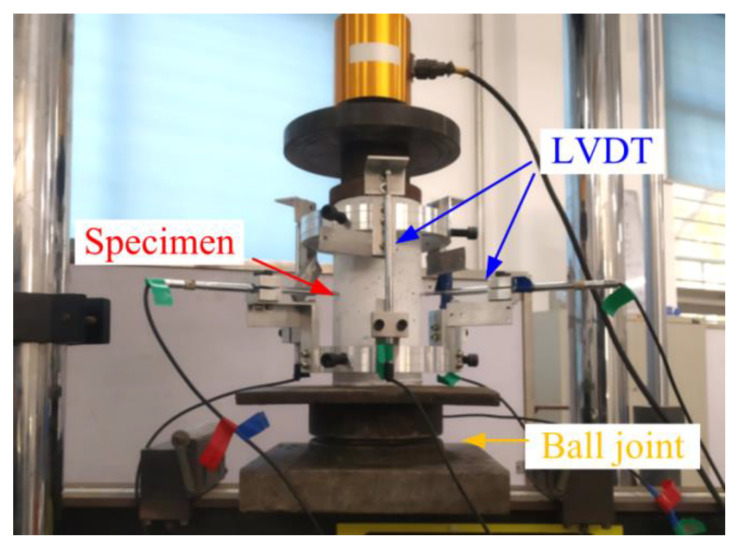
Volume measuring device under compressive load.

To examine the correlation between the permeability threshold and volume deformation, volume change measurements of concrete specimens were carried out under compressive loads. The experimental setup, as illustrated in Figure 5, was inspired by Choinska et al. [32]. The loading speed for the volume change test was similar to that for the permeability test. Moreover, due to the roughness of the specimen surface after freeze-thaw cycles, six linear variable differential transformers (LVDTs) were utilized to measure the deformation data under compressive load. Three of these LVDTs, which were positioned 120° relative to each other, were used to measure the radial deformation Δ*R*_0_ at the mid-height of the specimens. The remaining three LVDTs, which featured a gauge length of 175 mm and similarly arranged at 120° intervals, were used to assess the axial deformation Δ*l*. The volume of the hollow cylindrical specimens *V*, as derived from the radial deformation Δ*R*_0_ and axial deformation Δ*l*, is expressed in Equation (3).

(3) $$V=(l+\Delta l)\cdot \pi \cdot (R_{0}+\Delta R_{0}{)}^{2}$$

## 3. Results and Discussion

### 3.1. Test Results of Workability and Compressive Strength

Based on ASTM standards C143 [33] and C231 [34], the slump and air content of fresh PFRC were tested. Three specimens of different samples with a size of 150 mm × 150 mm × 150 mm were cast to evaluate the 28-day compressive strength. The slump, air content, and compressive strength are listed in Table 2.

As the PF content increased, a decrease in the slump and an increase in the air content of the concrete paste were observed. The compression test showed almost identical 28-day compressive strength of specimens with different PF dosages.

### 3.2. Concrete Permeability under Compressive Load

Considering that concrete structures typically experience compressive loads below peak values during normal usage, this study employed the compressive stress level (n) to analyze the effect of PF on the concrete permeability exposed to freeze-thaw cycles under compressive load, as expressed by Equation (4).

(4) $$n=\frac{F}{F_{p}}$$

where *F* is the compressive load of specimens (kN) and *F_p_* is the peak load of specimens (kN).

**Figure 6:**
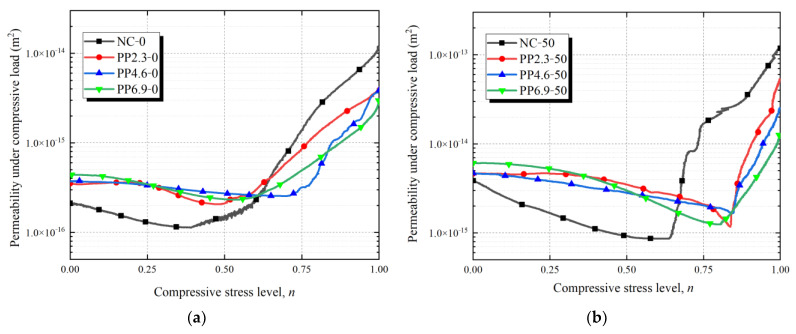

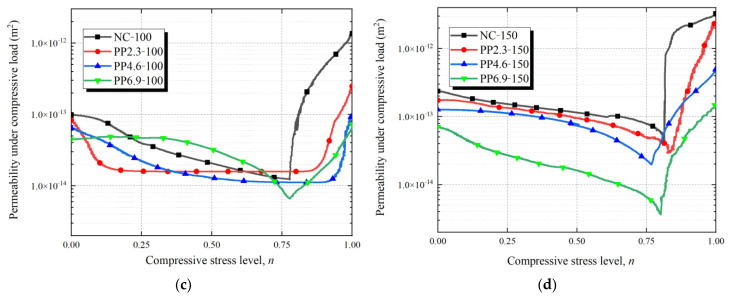
Relationship between compressive stress level and permeability of PFRC and NC exposed to freeze–thaw cycles: (**a**) 0 freeze–thaw cycle; (**b**) 50 freeze–thaw cycles; (**c**) 100 freeze–thaw cycles; (**d**) 150 freeze–thaw cycles.

Figure 6 depicts the relationship between the compressive stress level (ranging from 0 to 1) and the concrete permeability before the peak load. Table 3 presents the permeability and compressive stress levels at the minimum permeability points as well as the permeability at the peak load (*n* = 1) of PFRC under compressive load.

Based on Figure 6 and Table 3, for unfrozen PFRC, the initial permeability (permeability without load) was ranked as follows: NC-0 < PP2.3-0 < PP4.6-0 < PP6.9-0. This indicates that the incorporation of PF increases the permeability of the sound concrete matrix, and the permeability of PFRC increases with the fiber content. Similar results were obtained by Qin et al. [35], who explained that PF increase the internal porosity of concrete matrix, thereby enhancing the permeability. However, the initial permeability of the concrete subjected to 150 freeze-thaw cycles was ranked as follows: PP6.9-150 < PP4.6-150 < PP2.3-150 < NC-150. This is contrary to the initial permeability of the unfrozen concrete. Freeze-thaw damage increases internal damage and cracking in PFRC. Compared to NC specimens, PF can reduce freeze-thaw damage in the concrete matrix. Additionally, with high PF content, fewer micro-cracks form due to freeze-thaw damage, resulting in lower permeability in PFRC specimens.

Under compressive load, the compressive stress level-permeability curves of PFRC and NC can be classified into two stages. In stage I, the permeability of each specimen decreased as the compressive stress increased. As the compressive stress level continued to increase, the permeability of each specimen group increased, reaching a permeability threshold. The compressive stress level (*n_t_*) at the threshold point of compressive stress level-permeability curves between the first and second stages for PFRC specimens ranged from *n_t_* = 0.486 to 0.844 (approximately 0.75). Mehta et al. [36] found that above 75 percent of the ultimate load, very high strains develop with increasing stress, indicating that the crack system becomes continuous due to the rapid propagation of cracks in both the matrix and the interfacial transition zone.

Based on the above analysis, it is evident that in stage I, when *n* ≤ *n_t_*, the concrete is compressed under the compressive load, reducing the pore volume within the concrete matrix. This leads to a significant decrease in the permeability of PFRC during this stage, with the primary water pathways being internal pores and micro-cracks within the concrete matrix. In stage II, when *n* > *n_t_*, micro-cracks of concrete start to expand and interconnect as the compressive stress increases. Many microcracks form macrocracks, resulting in a significant increase in crack width. According to Poiseuille law [37], the permeability of cracks is proportional to the square of the crack width, which leads to a substantial increase of permeability of concrete with macro-cracks.

Compared with NC-0, the compressive stress levels of the minimum permeability points of PP2.3-0, PP4.6-0, and PP6.9-0 increased by 24.6%, 79.2%, and 38.7%, respectively. Compared with NC-50, the compressive stress levels of the minimum permeability points of PP2.3-50, PP4.6-50, and PP6.9-50 increased by 37.2%, 38.1% and 31.3%, respectively. Compared with NC-100, the compressive stress levels of the minimum permeability points of PP2.3-100, PP4.6-100, and PP6.9-100 increased by 28.3%, 36.3% and 27.5%, respectively. Compared with NC-150, the variations in the compressive stress level of the minimum permeability point of PP2.3-150, PP4.6-150, and PP6.9-150 were 1.6%, −5.8%, and −1.6%, respectively. For concrete without freeze-thaw damage, the stress levels corresponding to the permeability threshold of PFRC were significantly higher than those of NC. This indicates that PF can effectively limit the width of concrete cracks and increase the stress levels of macro-cracks formed under compressive load. As a result, concrete with PF can maintain lower permeability over a wider range of stress levels. However, with the increase of freeze-thaw cycles, the stress levels corresponding to the permeability thresholds of both NC and PFRC specimens increase, and the differences between them significantly decrease. Freeze-thaw cycles reduce the mechanical properties of concrete and increase the surface roughness of cracks, which decreases the crack permeability [38]. This might increase the stress level corresponding to the concrete permeability threshold and reduce the difference in compressive stress levels corresponding to the thresholds between PFRC and NC specimens.

At a constant number of freeze-thaw cycles, the permeability of the specimens at the peak load is ranked as follows: NC < PP2.3 < PP4.6 < PP6.9. This implies that the application of PF enhances the integrity of concrete specimens under peak loads, effectively increasing the roughness of concrete crack surfaces, and reduces the permeability of cracked concrete.

### 3.3. Volume Change of PFRC under Compressive Load

**Figure 7:**
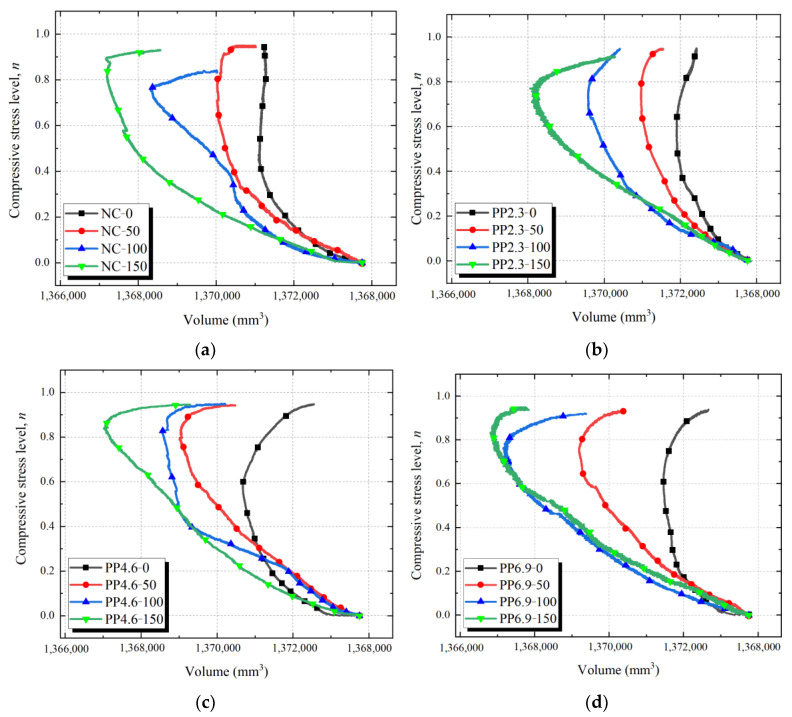
Relationship between compressive stress level and volume under compressive load of PFRC and NC: (**a**) NC specimens; (**b**) PP2.3 specimens; (**c**) PP4.6 specimens; (**d**) PP6.9 specimens.

Figure 7 illustrates the volume-compressive stress level curves for each specimen group. Because the measuring device and LVDTs were attached to the surface of the concrete specimens, numerous macro-cracks appeared as the compressive load approached the peak load of the concrete specimens. The macro-cracks decreased the stability of both the measuring device and LVDTs on the specimen surface. Thus, only the relationships between the specimen volume and compressive stress level before the peak load are presented here. Table 4 presents the minimum volumes recorded for each sample group and the corresponding compressive stress levels.

From Figure 7 and Table 4, the volume-compressive stress level curves of each specimen group exhibited two stages prior to the peak load. In stage I, the specimen volume decreased as the compressive stress level increased. Stage II began at the lowest volume of the concrete specimen; the volume of the specimens increases with the increasing of compressive stress level. According to the volume-compressive stress level curve, a threshold phenomenon is observed in the volume change of the specimens. The compressive stress level (*n_t_*’) corresponding to the threshold point of volume-compressive stress level curve for PFRC ranges from 0.562 to 0.837. The values of *n_t_*’ are close to those of *n_t_*.

As the freeze-thaw cycles increased, the compressive stress level of the minimum volume of PFRC and NC increased as well. Compared with NC-0, the increases in the compressive stress levels corresponding to the minimum volumes of NC-50, NC-100, and NC-150 were 56.8%, 69.3%, and 98.4%, respectively. Compared with PP2.3-0, the increases in the compressive stress levels corresponding to the minimum volumes of PP2.3-50, PP2.3-100, and PP2.3-150 were 29.7%, 30.8%, and 37.0%, respectively. Compared with PP4.6-0, the increases in the compressive stress levels corresponding to the minimum volumes of PP4.6-50, PP4.6-100, and PP4.6-150 were 37.5%, 42.7%, and 45.8%, respectively. Compared with PP6.9-0, the increases in the compressive stress levels corresponding to the minimum volumes of PP6.9-50, PP6.9-100, and PP6.9-150 were 20.8%, 23.5%, and 35.1%, respectively. An increase in the freeze-thaw cycles correlates with a consistent reduction in the minimum volume of all specimen groups. With the increase in freeze-thaw cycles, both PFRC and NC specimens can maintain the trend of decreasing volume with increasing stress levels over a wider range of compressive stress levels. This can be attributed to the increase in the pore content within the concrete and the formation of micro-cracks due to the freeze-thaw cycles. When the concrete specimen was subjected to compressive load, the volume compression of its pores and micro-cracks increased. At this time, the minimum volume of the concrete specimen with the same PF content decreased as freeze-thaw cycles increased. Furthermore, the increase in *n_t_*’ of NC specimen is more pronounced than those of PFRC specimens. For PF contents of 2.3 to 6.9 kg/m^3^ in the concrete specimens, when PFRC was exposed to the same freeze-thaw cycles, the differences in the minimum volumes (*V_min_*) of the specimens with different PF dosages were insignificant. It means that the volume change of PFRC did not significantly correlate with the PF contents. This indicates that the freeze-thaw cycles exert a greater effect on the volumetric deformation of PFRC than the dosage of PF.

### 3.4. Relationship of Compressive Stress Levels at the Minimum Permeability and the Minimum Volume Points of PFRC

**Figure 8:**
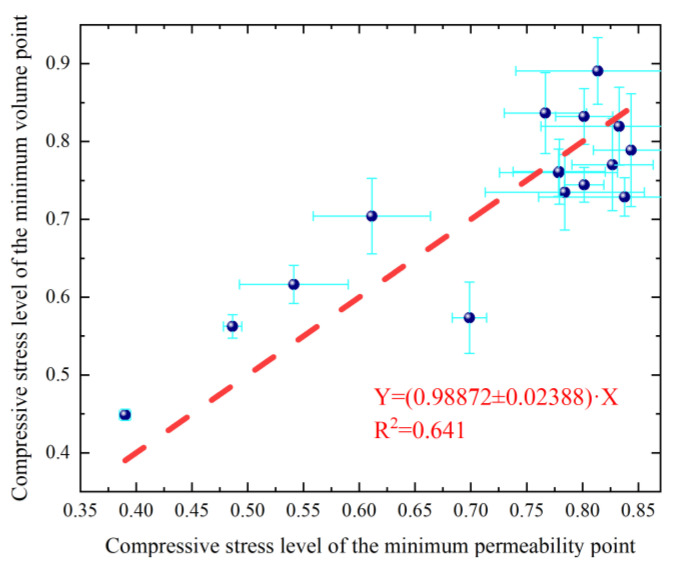
Relationship between compressive stress level of the minimum permeability point and compressive stress level of the minimum volume point of PFRC and NC (the purple circles are data points, and the red dot line is the fitted line of the data points.).

The relationship between the average compressive stress levels at the minimum permeability and the minimum volume points of PFRC is investigated and shown in Table 5. Figure 8 illustrates the relationship between the compressive stress levels at the minimum permeability points on the compressive stress level-permeability curves and those at the minimum volume points on the concrete volume-compressive stress level curves of PFRC. Their correlation was investigated using a proportional fitting function, as shown in Equation (5).

(5) Y=γ⋅X

As shown in Figure 8, the parameter *γ* of the proportional function fitted based on the experimental data is *γ* = 0.98872 ± 0.023. This indicates that *γ* ≈ 1 and the correlation coefficient *R*^2^ is 0.641. The different concrete specimens utilized in the permeability and mechanical tests may have contributed to the low *R*^2^ of the fitting equation. Considering this factor, the compressive stress levels at the minimum permeability points and the minimum volume points were almost equal and indicated a correlation. The result is similar to that found in the research by Xue et al. [39]. This indicates that, under compressive load, the volume of concrete decreased gradually as the compressive stress levels increased. As the pores within the concrete are compressed, this results in a gradual reduction in the permeability of the concrete matrix. When *n*_t_’ reached a certain compressive stress level of the minimum volume point, micro-cracks within the concrete matrix begin to expand and interconnect, forming macro-cracks [40]. This leads to an increase in the volume of the concrete specimens containing macro-cracks. The presence of macro-cracks significantly increases the permeability of the concrete. Changes in the porosity and micro-cracks within the concrete matrix affect the permeability performance of the concrete. Therefore, the compressive stress level at the minimum permeability point is related to that of the minimum volume point of each group under compressive load.

### 3.5. Permeability of Concrete under Tensile Load and Strain-Permeability Curve under Loading

**Figure 9:**
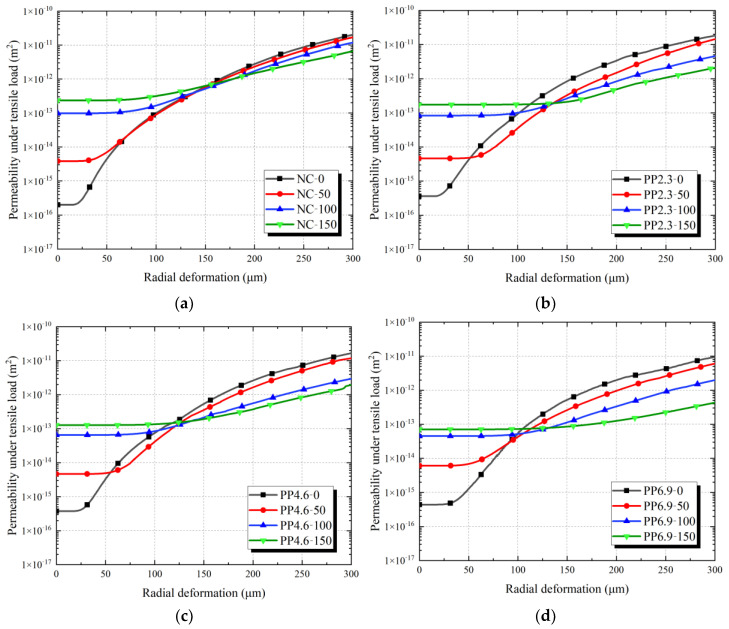
Relationship of permeability and radial deformation under tensile load of PFRC and NC: (**a**) NC specimens; (**b**) PP2.3 specimens; (**c**) PP4.6 specimens; (**d**) PP6.9 specimens.

Figure 9 illustrates the correlation between radial deformation and permeability of PFRC under tensile load. Table 6 presents a comparison of permeability values across various specimen groups under radial deformations of 0 and 200 μm. The *κ_T_*_−0_ and *κ_T−_*_200_ denote the permeability values at radial deformations of 0 and 200 μm under tensile load, respectively.

From Figure 9 and Table 6:

For concrete without freeze-thaw damage, an increase in the PF content corresponded to a decrease in the initial permeability. The initial permeability of PFRC and NC ranked as follows: NC-0 < PP2.3-0 < PP4.6-0 < PP6.9-0. As the freeze-thaw cycles increased, all specimens exhibited an increase in initial permeability. However, the rate of increase in the initial permeability was low for specimens with a high PF content. After 150 freeze-thaw cycles, the initial permeability order was as follows: PP6.9-150 < PP4.6-150 < PP2.3-150 < NC-150, which is consistent with the results in Section 3.2.

Under tensile load, the radial deformation-permeability curves of PFRC exhibited two stages. In stage I, as the radial deformation of the specimen intensified, the permeability of the concrete increased gradually, and the variance between the permeability of the specimen and its initial value was slight, which has been similarly reported in a previous study of Hubert et al. [41]. The increase in the permeability of PFRC is primarily caused by the generation of cracks in the concrete under tensile load. When the radial deformation of concrete is small, the concrete does not develop cracks under tensile load, or the crack widths are minimal, the permeability of concrete cracks is very low. Additionally, the rough crack surfaces significantly reduce the permeability of the cracks [42]. In this stage, permeation in the specimens primarily occurred through the inherent porous channels of the concrete matrix. Therefore, it exhibited a trend similar to that of the initial permeability of PFRC. The experimental data indicate that the tensile load minimally affects the porous channels of the concrete matrix, as supported by [43]. 

In the second stage of the radial deformation-permeability curves under tensile load, the concrete permeability increased significantly with the radial deformation of PFRC. As the freeze-thaw cycles increased, the corresponding permeability value for PFRC decreased gradually. Additionally, a high PF content correlated with a significant reduction in the permeability of PFRC. For instance, compared with PP4.6-0, the permeability values *κ_T−_*_200_ of PP4.6-50, PP4.6-100, and PP4.6-150 decreased by 36.8%, 78.5%, and 85.8%, respectively. For PP6.9, compared with PP6.9-0, the permeability values *κ_T−_*_200_ of PP6.9-50, PP6.9-100, and PP6.9-150 decreased by 51.5%, 83.2%, and 93.7%, respectively. This decrease in the permeability of PFRC was attributed to the propagation of cracks in the concrete specimens as the radial deformation increased. The water channels in these cracked specimens included the porous concrete matrix and the concrete cracks. The crack permeability was significantly higher than that of a sound concrete matrix [19]. As the radial deformation of PFRC increased, the crack width expanded, resulting in a high concrete permeability growth rate. Yi et al. [42] also observed similar phenomena and found that the permeability of concrete increases significantly when the crack width reaches 50 μm.

Karahan et al. [43] reported that PF limited crack development and the expansion of the concrete matrix under freeze-thaw conditions, and the higher the PF content, the higher the freeze resistance of PFRC. The addition of PF resulted in a rough crack surface on the specimens. Meanwhile, freeze-thaw cycles increase the damage and porosity of concrete matrix, resulting in a rough crack surface under tensile load [23]. For PFRC subjected to freeze-thaw cycles, rough crack surfaces and multi-branched cracks significantly reduce its permeability. Consequently, the head loss across the cracks becomes significant, reducing the permeability of the cracks. Therefore, compared to PFRC without freeze-thaw damage, PFRC with freeze-thaw damage exhibits better impermeability. This similar finding is corroborated by the research conducted by Meng et al. [44]. 

**Figure 10:**
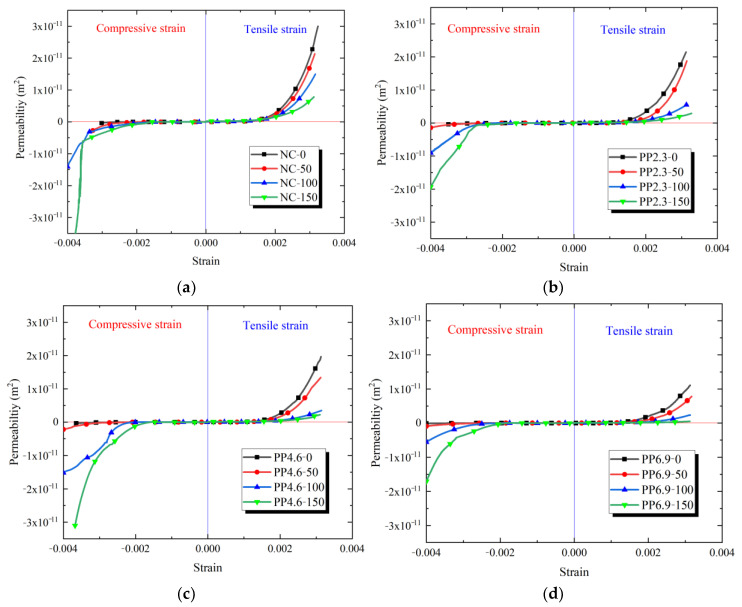
Strain-permeability curves under splitting tension and compressive loads of PFRC and NC: (**a**) NC specimens; (**b**) PP2.3 specimens; (**c**) PP4.6 specimens; (**d**) PP6.9 specimens.

To compare the effects of compressive and tensile loads on the permeability of PFRC specimens, Figure 10 illustrates the relationship between strain and permeability of PFRC subjected to compressive and tensile loads. When the strain and permeability values are positive, the curve shows the relationship between permeability and tensile strain of PFRC. When the strain and permeability values are negative, the curve shows the relationship between permeability and compressive strain of PFRC.

Figure 10 illustrates the concrete permeability of PFRC under compressive and tensile loads. The curves are classified into two stages. In stage I, the permeability of PFRC increased slightly with the tensile strain, whereas it decreased gradually as the compressive strain increased. In this stage, under both compressive and tensile loads, the permeability of PFRC shows opposite trends with increasing strain.

Meanwhile, the following were observed in stage II:

(i) The concrete permeability of PFRC increased significantly with the increase in both compressive and tensile strains.
(ii) For the specimens subjected to the same number of freeze-thaw cycles, the permeability of PFRC decreased gradually as the PF content increased.
(iii) Under tensile load, as the freeze-thaw cycles increased, the permeability of PFRC decreased gradually. Conversely, as the freeze-thaw cycles increased, the permeability of PFRC increased gradually under compressive load. Under compressive and tensile loads, the permeability trends of PFRC were diametrically opposed.

As discussed previously, in stage II of the tensile strain-permeability curves, the water paths of PFRC included both the concrete matrix and cracks. The permeability of the concrete matrix was lower than that of concrete cracks. During this stage, the crack permeability was predominant. The PF inhibited the propagation of concrete cracks. Simultaneously, the application of PF and freeze-thaw cycles intensified the roughness of the crack surface and subsequently decreased the permeability of PFRC under tensile strain [6].

For the second stage of the compressive strain-permeability curves, an increase in freeze-thaw cycles resulted in the formation of multiple wide cracks in the PFRC. The compressive load was mainly supported by the concrete matrix. The freeze-thaw damage of PFRC significantly decreased its compressive load-bearing ability and increased its brittleness. Consequently, an increase in the freeze-thaw cycles significantly increased the permeability of concrete under compressive loads. In stage II, under both compressive and tensile loads, the permeability of PFRC exhibited opposite trends with increasing number of freeze-thaw cycles.

Based on the analysis above, the effect of PF on limiting the concrete permeability subjected to tensile load was greater than that subjected to compressive load. In future research, the relationship between strain and permeability of PFRC can be integrated with its constitutive relationship between stress and strain to provide a reference for the application of PF in the waterproofing of concrete structures.

## 4. Conclusions

Based on the experimental and analytical results, the following conclusions can be drawn:

(1) The application of PF increases the porosity of the concrete matrix, negatively affecting the impermeability of the undamaged concrete matrix. However, as the freeze-thaw cycles increase, PF can effectively reduce freeze-thaw damage, resulting in a slower increase in the initial permeability of PFRC compared to NC. The addition of PF demonstrates a positive effect on the impermeability of concrete subjected to freeze-thaw cycles.
(2) Under compressive load, as the stress level increases, the permeability of polypropylene fiber reinforced concrete (PFRC) initially shows a slow decreasing trend, followed by a significant increase. A permeability threshold is observed and divides the evolution of PFRC permeability into two stages. The compressive stress levels at the points of minimum permeability and minimum volume for PFRC exhibit a linear correlation, with the fitted proportional function parameter *γ* = 0.98872 ± 0.023. This permeability trend is related to the volume changes of the specimens under compressive load. For concrete without freeze-thaw damage, the incorporation of PF enables the concrete to maintain lower permeability over a wider range of compressive stress levels. As the freeze-thaw cycles increase, the threshold stress levels for permeability in both NC and PFRC increase and tend to become similar, extending the range of compressive stress levels that maintain low permeability.
(3) Under tensile load, the radial deformation-permeability curves of PFRC exhibit two stages. In stage I, the permeability of PFRC evolves with increasing radial deformation, with the concrete matrix being the primary water pathway. This results in a relatively small increase in permeability. In stage II, as the radial deformation increases, the crack width in the concrete gradually enlarges, leading to a significant increase in the permeability of PFRC. The addition of PF and the occurrence of freeze-thaw cycles both reduce the permeability of concrete in stage II of the radial deformation-permeability curves under tensile load.
(4) The relationship between strain and permeability of PFRC under both compressive and tensile loads is determined, and it comprises two stages. In stage I, when compressive and tensile strains are low, the permeability of PFRC does not change significantly. The permeability of PFRC gradually increases with increasing tensile strain, while it gradually decreases with increasing compressive strain. Thus, in stage I, the permeability trends of PFRC under compressive and tensile loads are opposite as the strain increases. In stage II, when tensile and compressive strains are high, the permeability of PFRC increases significantly. Under tensile load, the permeability of PFRC decreases with the increasing in freeze-thaw cycles, while under compressive load, the permeability gradually increases with an increasing of freeze-thaw cycles. In stage II, the permeability trends of PFRC under compressive and tensile loads are completely opposite as the freeze-thaw cycles increase. The promotion in concrete impermeability due to the polypropylene fiber subjected to tensile load was larger than that subjected to compressive load. In future research, the relationship between strain and permeability of PFRC can be integrated with its constitutive relationship between stress and strain to provide a reference for the application of PF in the waterproofing of concrete structures.

## Figures and Tables

**Table 1:** Mix proportions of different samples.

Mixture ID	Polypropylene Fiber (kg/m^3^)	Portland Cement (kg/m^3^)	Fine Aggregate (kg/m^3^)	Coarse Aggregate (kg/m^3^)	Water (kg/m^3^)	Fly Ash (kg/m^3^)	Superplasticizer (kg/m^3^)
NC	--	390	822	848	272.5	155	4.0
PP2.3	2.3 (0.25 vol.%)	390	822	848	272.5	155	4.0
PP4.6	4.6 (0.50 vol.%)	390	822	848	272.5	155	4.0
PP6.9	6.9 (0.75 vol.%)	390	822	848	272.5	155	4.0

**Table 2:** Slump, air content and compressive strength of NC and PFRC.

Sample	Slump (mm)	Air Content (%)	28-Day Compressive Strength (MPa)
NC	195	1.9	37.3
PP2.3	192	2.2	36.5
PP4.6	185	2.5	35.4
PP6.9	170	2.7	37.5

**Table 3:** Comparison of minimum permeability, compressive stress level of the minimum permeability point, and the permeability of peak load of PFRC.

Sample	Minimum Permeability (m^2^)	Compressive Stress Level of the Minimum Permeability Point	Permeability of Peak Load (m^2^)	Sample	Minimum Permeability (m^2^)	Compressive Stress Level of the Minimum Permeability Point	Permeability of Peak Load (m^2^)
NC-0	1.13 × 10^−16^ (*C*v = 5.1%)	0.390 (*C*v = 1.3%)	1.193 × 10^−14^ (*C*v = 8.6%)	NC-100	1.23 × 10^−14^ (*C*v = 8.9%)	0.779 (*C*v = 6.8%)	1.36 × 10^−12^ (*C*v = 5.3%)
PP2.3-0	2.07 × 10^−16^ (*C*v = 7.9%)	0.486 (*C*v = 1.7%)	4.090 × 10^−15^ (*C*v = 6.1%)	PP2.3-100	1.59 × 10^−14^ (*C*v = 9.5%)	0.784 (*C*v = 9.1%)	2.48 × 10^−13^ (*C*v = 4.8%)
PP4.6-0	2.55 × 10^−16^ (*C*v = 4.4%)	0.699 (*C*v = 2.2%)	3.860 × 10^−15^ (*C*v = 4.7%)	PP4.6-100	1.12 × 10^−14^ (*C*v = 7.5%)	0.833 (*C*v = 8.4%)	9.23 × 10^−14^ (*C*v = 6.2%)
PP6.9-0	2.32 × 10^−16^ (*C*v = 9.9%)	0.541 (*C*v = 9.0%)	2.977 × 10^−15^ (*C*v = 9.3%)	PP6.9-100	6.59 × 10^−15^ (*C*v = 8.9%)	0.779 (*C*v = 5.3%)	6.39 × 10^−14^ (*C*v = 2.7%)
NC-50	8.65 × 10^−16^ (*C*v = 9.7%)	0.611 (*C*v = 8.6%)	1.188 × 10^−13^ (*C*v = 7.2%)	NC-150	4.43 × 10^−14^ (*C*v = 5%)	0.814 (*C*v = 9.0%)	3.28 × 10^−12^ (*C*v = 2.9%)
PP2.3-50	1.18 × 10^−15^ (*C*v = 5.5%)	0.838 (*C*v = 9.2%)	5.333 × 10^−14^ (*C*v = 1.6%)	PP2.3-150	2.92 × 10^−14^ (*C*v = 3.6%)	0.827 (*C*v = 4.4%)	2.33 × 10^−12^ (*C*v = 2.6%)
PP4.6-50	1.66 × 10^−15^ (*C*v = 2.3%)	0.844 (*C*v = 4.0%)	2.517 × 10^−14^ (*C*v = 1.2%)	PP4.6-150	1.98 × 10^−14^ (*C*v = 8.5%)	0.767 (*C*v = 4.8%)	4.93 × 10^−13^ (*C*v = 9.0%)
PP6.9-50	1.25 × 10^−15^ (*C*v = 6.1%)	0.802 (*C*v = 2.2%)	1.260 × 10^−14^ (*C*v = 3.0%)	PP6.9-150	3.63 × 10^−15^ (*C*v = 9.9%)	0.801 (*C*v = 3.2%)	1.47 × 10^−13^ (*C*v = 3.2%)

**Table 4:** Comparison of minimum volume and the corresponding compressive stress level of PFRC.

Sample	Minimum Volume of Specimen (mm^3^)	Compressive Stress Level	Sample	Minimum Volume of Specimen (mm^3^)	Compressive Stress Level
NC-0	1,371,085 (*C*v = 7.2%)	0.449 (*C*v = 1.6%)	NC-100	1,368,312 (*C*v = 1.5%)	0.760 (*C*v = 4.0%)
PP2.3-0	1,371,894 (*C*v = 5.3%)	0.562 (*C*v = 2.7%)	PP2.3-100	1,369,571 (*C*v = 2.0%)	0.735 (*C*v = 6.6%)
PP4.6-0	1,370,675 (*C*v = 3.4%)	0.574 (*C*v = 8.0%)	PP4.6-100	1,368,551 (*C*v = 7.2%)	0.819 (*C*v = 6.1%)
PP6.9-0	1,371,452 (*C*v = 1.8%)	0.616 (*C*v = 4.0%)	PP6.9-100	1,367,150 (*C*v = 5.2%)	0.761 (*C*v = 5.5%)
NC-50	1,370,018 (*C*v = 5.6%)	0.704 (*C*v = 6.9%)	NC-150	1,367,169 (*C*v = 1.8%)	0.891 (*C*v = 4.8%)
PP2.3-50	1,370,959 (*C*v = 5.0%)	0.729 (*C*v = 3.4%)	PP2.3-150	1,368,052 (*C*v = 4.6%)	0.770 (*C*v = 9.9%)
PP4.6-50	1,369,009 (*C*v = 4.2%)	0.789 (*C*v = 9.2%)	PP4.6-150	1,367,017 (*C*v = 4.5%)	0.837 (*C*v = 6.2%)
PP6.9-50	1,369,187 (*C*v = 7.7%)	0.744 (*C*v = 3.0%)	PP6.9-150	1,366,805 (*C*v = 5.4%)	0.832 (*C*v = 4.3%)

**Table 5:** Comparison of compressive stress level of the minimum permeability point and compressive stress level of the minimum volume point of PFRC.

Sample	Compressive Stress Level		Sample	Compressive Stress Level	
	Minimum Permeability Point	Minimum Volume Point		Minimum Permeability Point	Minimum Volume Point
NC-0	0.390 (*C*v = 1.3%)	0.449 (*C*v = 1.6%)	NC-100	0.779 (*C*v = 6.8%)	0.760 (*C*v = 4.0%)
PP2.3-0	0.486 (*C*v = 1.7%)	0.562 (*C*v = 2.7%)	PP2.3-100	0.784 (*C*v = 9.1%)	0.735 (*C*v = 6.6%)
PP4.6-0	0.699 (*C*v = 2.2%)	0.574 (*C*v = 8.0%)	PP4.6-100	0.833 (*C*v = 8.4%)	0.819 (*C*v = 6.1%)
PP6.9-0	0.541 (*C*v = 9.0%)	0.616 (*C*v = 4.0%)	PP6.9-100	0.779 (*C*v = 5.3%)	0.761 (*C*v = 5.5%)
NC-50	0.611 (*C*v = 8.6%)	0.704 (*C*v = 6.9%)	NC-150	0.814 (*C*v = 9.0%)	0.891 (*C*v = 4.8%)
PP2.3-50	0.838 (*C*v = 9.2%)	0.729 (*C*v = 3.4%)	PP2.3-150	0.827 (*C*v = 4.4%)	0.770 (*C*v = 9.9%)
PP4.6-50	0.844 (*C*v = 4.0%)	0.789 (*C*v = 9.2%)	PP4.6-150	0.767 (*C*v = 4.8%)	0.837 (*C*v = 6.2%)
PP6.9-50	0.802 (*C*v = 2.2%)	0.744 (*C*v = 3.0%)	PP6.9-150	0.801 (*C*v = 3.2%)	0.832 (*C*v = 4.3%)

**Table 6:** Comparison of permeability of PFRC and NC under tensile load.

Sample	*κ_T−_*_0_ (m^2^)	*κ_T−_*_200_ (m^2^)	Sample	*κ_T−_*_0_ (m^2^)	*κ_T−_*_200_ (m^2^)
NC-0	2.022 × 10^−16^ (*C*v = 3.1%)	2.791 × 10^−12^ (*C*v = 3.0%)	NC-100	9.880 × 10^−14^ (*C*v = 7.9%)	1.731 × 10^−12^ (*C*v = 1.9%)
PP2.3-0	3.656 × 10^−16^ (*C*v = 6.1%)	3.386 × 10^−12^ (*C*v = 9.5%)	PP2.3-100	8.371 × 10^−14^ (*C*v = 5.9%)	8.389 × 10^−13^ (*C*v = 2.4%)
PP4.6-0	3.760 × 10^−16^ (*C*v = 4.1%)	2.594 × 10^−12^ (*C*v = 8.7%)	PP4.6-100	6.493 × 10^−14^ (*C*v = 9.5%)	5.576 × 10^−13^ (*C*v = 8.0%)
PP6.9-0	4.410 × 10^−16^ (*C*v = 8.0%)	1.980 × 10^−12^ (*C*v = 2.4%)	PP6.9-100	4.492 × 10^−14^ (*C*v = 9.3%)	3.327 × 10^−13^ (*C*v = 3.5%)
NC-50	3.880 × 10^−15^ (*C*v = 5.9%)	2.331 × 10^−12^ (*C*v = 2.5%)	NC-150	2.371 × 10^−13^ (*C*v = 6.1%)	1.507 × 10^−12^ (*C*v = 6.7%)
PP2.3-50	4.640 × 10^−15^ (*C*v = 4.7%)	1.496 × 10^−12^ (*C*v = 8.0%)	PP2.3-150	1.740 × 10^−13^ (*C*v = 5.7%)	4.881 × 10^−13^ (*C*v = 8.7%)
PP4.6-50	4.700 × 10^−15^ (*C*v = 7.1%)	1.639 × 10^−12^ (*C*v = 7.4%)	PP4.6-150	1.274 × 10^−13^ (*C*v = 8.1%)	3.685 × 10^−13^ (*C*v = 3.8%)
PP6.9-50	6.120 × 10^−15^ (*C*v = 7.6%)	9.604 × 10^−13^ (*C*v = 1.3%)	PP6.9-150	7.050 × 10^−14^ (*C*v = 8.3%)	1.257 × 10^−13^ (*C*v = 8.1%)

## Data Availability

Data are contained within the article.
